# Immunogenicity, safety and reactogenicity of a Phase II trial of Vi-DT typhoid conjugate vaccine in healthy Filipino infants and toddlers: A preliminary report

**DOI:** 10.1016/j.vaccine.2019.09.074

**Published:** 2020-06-09

**Authors:** Maria Rosario Capeding, Edison Alberto, Arijit Sil, Tarun Saluja, Samuel Teshome, Deok Ryun Kim, Ju Yeon Park, Jae Seung Yang, Suchada Chinaworapong, Jiwook Park, Sue-Kyoung Jo, Yun Chon, Seon-Young Yang, Dong Soo Ham, Ji Hwa Ryu, Julia Lynch, Jerome H. Kim, Hun Kim, Jean-Louis Excler, T. Anh Wartel, Sushant Sahastrabuddhe

**Affiliations:** a Research Institute for Tropical Medicine, Manila, Philippines; b International Vaccine Institute, Seoul, Republic of Korea; c SK Bioscience, Seoul, Republic of Korea

**Keywords:** Typhoid fever, Typhoid conjugate vaccine, Vi-DT, Immunogenicity, Safety, Infants, Toddlers, Philippines

## Abstract

**Background:**

Typhoid fever remains an important public health problem in developing countries and is endemic in many parts of Asia and Africa where the incidence of disease typically peaks in school-aged children. Age restrictions and other limitations of existing oral live-attenuated typhoid and parenteral Vi polysaccharide vaccines have triggered the development of Vi conjugate vaccines with improved immunological properties, use in younger age range, and longer durability of protection. We present the safety, reactogenicity, and immunogenicity data from a Phase II study after a single dose of Vi polysaccharide conjugated to diphtheria toxoid (Vi-DT) conducted in 6–23-month old Filipino children.

**Methods:**

This is a randomized, observer-blinded Phase II study to assess the immunogenicity, safety and reactogenicity of Vi-DT compared to placebo, conducted in Muntinlupa City, The Philippines. Participants aged 6–23 months were enrolled and randomized to Vi-DT (25 µg) or placebo (0.9% sodium chloride) and evaluated for immunogenicity and overall safety 28 days post vaccination.

**Results:**

A total of 285 participants were enrolled and age-stratified: 6 to < 9 months, 9–12 months, and 13–23 months. Seventy-six (76) participants received Vi-DT and 19 received placebo per each strata. All participants seroconverted after a single dose of Vi-DT versus 7% of placebo recipients. Anti-Vi IgG GMT was 444.38 [95% CI (400.28; 493.34)] after a single dose of Vi-DT; there was no change in GMT after placebo administration, 0.41 [95% CI (0.33; 0.51), p < 0.0001]. A similar pattern of immunogenicity was reported across all age strata. The vaccine reported to be safe and well tolerated.

**Conclusions:**

Vi-DT vaccine was immunogenic, safe, and well tolerated in children aged 6–23 months.

ClinicalTrials.gov registration number: NCT03527355.

## Highlights

• Vi-DT is safe and well tolerated in infants and toddlers aged 6–23 months.
• Vi-DT showed >1000-fold higher Geometric Mean Titer (GMT) compared to Placebo.
• 100% seroconversion reported in Vi-DT group in all age strata.

## 1 Introduction

Typhoid fever, an invasive bacterial infection caused by *Salmonella enterica serovar* Typhi (*S*. Typhi), remains an important public health problem, especially in low- and middle-income countries. It is a common cause of bacteremia in many resource-poor settings, with most of the cases being reported from the Indian subcontinent of South Asia [1]. Global estimates suggest that there are approximately 10.9 million cases and 116.8 thousand deaths annually from typhoid [2]. Although the information of typhoid fever burden is scattered, typhoid is endemic in the Philippines [3]. According to the Department of Health, a total 22,234 suspected typhoid cases were reported nationwide from Jan 1 to Dec 3, 2018 [4].

All age groups are prone to typhoid infection but school-aged children (5–15 years old) are most significantly impacted. In some endemic areas, children under 5 years of age have incidence rates similar to or exceeding those of school-aged children [5], [6], [7], [8], [9], [10], [11], [12], [13]. A systematic review reported that a median of 58% of typhoid fever episodes occurred in children <5 years old [9]. Typhoid fever surveillance or epidemiology studies carried out between 1998 and 2017 in Africa, Asia, and the Americas with >10,000 blood culture-confirmed cases of typhoid fever sufficiently severe to require outpatient or inpatient care showed that 27% of all cases occurred in children aged 0–4 years; 30% of those cases occurred in children aged <2 years and 10% in infants aged <1 year [5].

Although typhoid fever can be treated with antibiotics, the emergence of multidrug resistant (MDR) and extremely drug resistant (XDR) strains of *S*. Typhi has important and unfortunate implications. Resistance to the traditional first-line antibiotics ampicillin, chloramphenicol, and trimethoprim sulfamethoxazole developed in the late 1980s, then fluoroquinolone resistance in the 1990s, and more recently extended to cephalosporins have emerged in Asia and Africa [14], [15], [16], [17], [18], [19], [20].

The World Health Organization (WHO) cited the increasing problem of antibiotic-resistant *S*. Typhi as one of the key rationales for typhoid fever vaccination in populations at high risk of infection. The prevention of typhoid fever through immunization and other measures has the potential to decrease antibiotic use and limit the emergence of resistant *S*. Typhi [5], [21], providing a short-to-medium term measure to lower typhoid disease burden [22].

Typhoid vaccines licensed and recommended for use are live attenuated Ty21a, Vi polysaccharide, and typhoid conjugate vaccines (TCV) [21]. TCV is preferred over oral and polysaccharides vaccines because of its improved immunological properties, suitability for use in younger children, and longer duration of protection [23]. Vi polysaccharide and conjugate vaccines are recommended by WHO for the control of typhoid in endemic and epidemic settings along with water, sanitation and hygiene (WASH) intervention. WHO recommends that the introduction of TCV should be prioritized in countries having high burden of typhoid disease [21].

Three Vi polysaccharide vaccines conjugated to tetanus toxoid as carrier protein are licensed in India for use from 6 months of age. Typbar-TCV® (Bharat Biotech International Ltd) was prequalified by WHO in January 2018 [24]. The International Vaccine Institute (IVI) in collaboration with SK bioscience developed a typhoid conjugate vaccine consisting of Vi polysaccharide [India clinical isolate (C6524)] conjugated to diphtheria toxoid. Vi-DT was safe, well tolerated, and immunogenic in a Phase I clinical trial carried out in 144 healthy Filipino subjects aged 2–45 years [23]. This report provides information on the immunogenicity, safety, and tolerability of a single dose of Vi-DT from a Phase II trial in children 6–23 months of age.

## 2 Materials and methods

This phase II clinical trial (Clinicaltrials.gov NCT03527355) was approved by the Philippines Food and Drug Administration (PFDA) and the Institutional Review Boards (IRB) of the Research Institute for Tropical Medicine (RITM) and IVI. The study was conducted in compliance with International Council for Harmonization (ICH) Good Clinical Practice (GCP) E6 (R2) and E11, Belmont Principles, CIOMS guidelines, Declaration of Helsinki and other applicable regulations in the Republic of the Philippines. Before study participation, written informed consent was obtained from the parents or legal guardians of trial participants.

### 2.1 Study design and participants

This is a randomized, observer-blinded (safety evaluators and other trial staff remained blinded with the exception of the vaccine administrator), phase II study in healthy infants and toddlers aged 6–23 months at study initiation. Eligible participants enrolled into the study were randomized into one of three groups (Groups A, B, and C). The study was further age-stratified within each group: 6 to less than 9 months, 9–12 months, and 13 to 23 months. Groups A and B received a single dose of Vi-DT (25 μg) and Group C received a single dose of placebo. MMR was co-administered to those 9–12 months of age. Blood samples were collected prior to vaccination and 4 weeks post investigational product (IP) administration for immunogenicity assessment. At 24 weeks, Groups A and C will receive a non-typhoid vaccine while Group B will receive a second dose of Vi-DT.

The study evaluates primary immunogenicity endpoint which is the rate of anti-Vi seroconversion in age-stratified recipients after a single dose of Vi-DT or placebo. For the purpose of this report we will refer to Groups A and B combined as the Vi-DT group and Group C as placebo group.

This pre-specified interim analysis was performed after all participants completed the week 4 visit in order to facilitate Phase 3 planning. The immunogenicity and safety data through 4 weeks of study were cleaned, locked, and un-blinded for analysis. This interim analysis was performed by an independent statistician who was not directly involved in the study and study personnel remain blinded to the allocation of test vaccine and placebo.

### 2.2 Investigational product

The Vi-DT vaccine contains two active ingredients, 25 µg of purified Vi polysaccharide (*S.* Typhi C6524) conjugated with diphtheria toxoid (*Corynebacterium diphtheria* PW No.8) and formulated with stabilizers. It is presented in liquid form at 0.5 mL per glass vial. Placebo was 0.5 mL of 0.9% sodium chloride isotonic solution (EuroMed Inc, Manila, Philippines). Vi-DT and Placebo were given intramuscularly into the anterolateral left thigh of the infants and toddlers. IP (Test vaccine/Placebo) were stored at +2–8 °C at the RITM facility.

### 2.3 Assessment of safety and reactogenicity

Immediate reactions were assessed at the clinical trial site for 60 min following vaccination. Parents/guardians were provided with a thermometer and diary cards (DC) to record axillary temperature and any adverse events (AE) daily: solicited AE for 7 days after vaccination and unsolicited AE for 28 days. Solicited local adverse reactions included pain, tenderness, erythema/redness, swelling/induration and pruritus at the injection site. Solicited systemic adverse reactions included fever, lethargy, irritability, vomiting, diarrhea, drowsiness, loss of appetite, and persistent crying. Rash and nasopharyngitis were described in protocol as possible MMR vaccine-specific adverse events and were collected for 7 days following the vaccine administration for the toddlers aged 9 to less than 12 months. Unsolicited Adverse Events were defined as, any adverse event not specifically documented among the solicited AE that occurred from the date of administration of the test vaccine or placebo from days 0–28. Unsolicited AEs were classified into System Organ Class (SOC) and Preferred Term (PT) using MedDRA (version 21, 2018). Participants were invited to visit the study site on Days 3, 7, and 28 after injection for safety assessment and in case of development of any AE. Serious adverse events (SAE) were recorded during the entire study period. Blinded safety data were regularly reviewed by the sponsor’s Safety Monitoring Committee (SMC).

### 2.4 Assessment of immunogenicity

Blood serum samples from baseline and 28 days post injection were stored at −20 °C −80 °C until analysis. An anti-Vi IgG ELISA was used to measure anti-Vi specific antibodies of the IgG isotype in human sera as previously described [23], [25].

The level of the specific anti-Vi IgG as international unit (IU) units for each serum sample was determined by comparison to a reference serum (NIBSC 16/138).

### 2.5 Statistical analysis

The primary immunogenicity endpoint was measured as seroconversion rate at week 4. Seroconversion rate was defined as the proportion of participants with at least 4-fold rise anti-Vi IgG ELISA antibody titers (with 95% CI for each group) at week 4 as compared to baseline (Day 0). The analysis of the primary endpoint was done by the stratified Cochran-Mantel-Haenszel (CMH) test.

This endpoint was tested at the interim analysis with type 1 error rate of 0.0125 (one-sided test from stratified CMH test) to control overall study wise type 1 error rate of 0.025. An analysis of covariance model was performed as a sensitivity analysis in examining the differences of GMT and GM fold rise that are related to the effect of controlled baseline characteristics.

The immunogenicity set was defined as participants who were randomized, received at least one dose of IP and provided at least one post baseline measure for immunogenicity.

All randomized participants were included in the analysis of demographics and baseline characteristics. Demographic characteristics of continuous variables were summarized by number of participants, mean, standard deviation, median, minimum and maximum, and categorical variables were summarized by frequency and percentage in each vaccine group.

Safety was assessed for all randomized participants who received at least one vaccination. The proportion of participants who experienced solicited and unsolicited AEs after dose was provided with the 95% CI of the proportion. The incidence of solicited and unsolicited AEs by severity, causality, and outcome was presented with proportion of participants.

Assuming 10% dropout rate, the sample size of n = 228 for Vi-DT group vs. n = 57 Placebo group provided >99% power to detect the superiority of seroconversion rate in Vi-DT regimen compared to Placebo with one-sided test at 0.0125 significance level. All analyses were performed using SAS 9.4 (SAS Institute, Cary NC).

## 3 Results

### 3.1 Study population

**Fig. 1:**
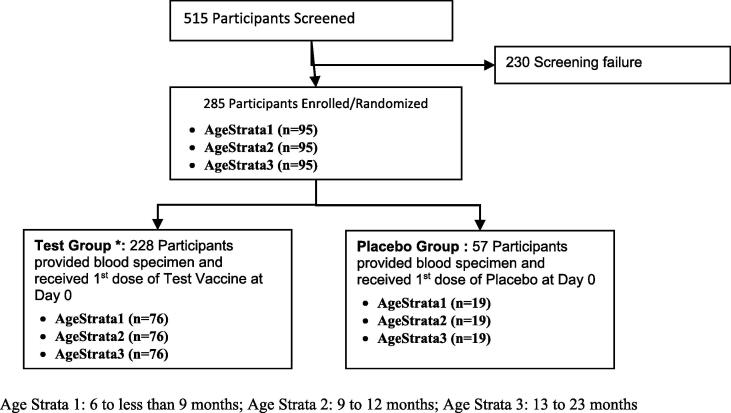
Flow diagram of Participant Disposition (CONSORT flow diagram). Age Strata 1: 6 to less than 9 months; Age Strata 2: 9–12 months; Age Strata 3: 13–23 months.

Among 515 participants screened, 285 participants were enrolled and randomized to either Test vaccine or Placebo at a 4:1 ratio. The most frequently reported cause of screen failure was related to health condition of potential participants (i.e., acute illness, abnormal clinical exam, lab values abnormalities). Of those randomized, 228 (100%) in Test and 57 (100%) in Placebo group completed the first dose administration and the 4-week follow-up (Fig. 1) and were included in this analysis. Both groups were comparable with regard to demographic and baseline characteristics (Table 1).

**Table 1:** Demographic characteristics of the study population.

Characteristics		Vi-DT Group	Placebo Group	Total
Overall		N = 228	N = 57	N = 285
Gender	Male (%)	109 (47.8)	29 (50.9)	138 (48.42)
	Female (%)	119 (52.2)	28 (49.1)	147 (51.58)

Age (months)	Mean (SD)	11.56 (5.45)	11.35 (5.14)	11.52 (5.38)
	Median (min, max)	9 (6, 23)	9 (6, 23)	9 (6, 23)

**6 to less than 9 months**		**N = 76**	**N = 19**	**N = 95**
Gender	Male (%)	32 (42.1)	11 (57.9)	43 (45.26)
	Female (%)	44 (57.9)	8 (42.1)	52 (54.74)

Age (months)	Mean (SD)	6.83 (0.87)	6.79 (0.71)	6.82 (0.84)
	Median (min, max)	7 (6, 8)	7 (6, 8)	7 (6, 8)

**9 to 12 months**		**N = 76**	**N = 19**	**N = 95**
Gender	Male (%)	39 (51.3)	9 (47.4)	48 (50.53)
	Female (%)	37 (48.7)	10 (52.6)	47 (49.47)

Age (months)	Mean (SD)	9.25 (0.64)	9.42 (0.96)	9.28 (0.71)
	Median (min, max)	9 (8, 12)	9 (9, 12)	9 (8, 12)

**13 to 23 months**		**N = 76**	**N = 19**	**N = 95**
Gender	Male (%)	38 (50.0)	9 (47.4)	47 (49.47)
	Female (%)	38 (50.0)	10 (52.6)	48 (50.53)

Age (months)	Mean (SD)	18.59 (3.24)	17.84 (3.24)	18.44 (3.24)
	Median (min, max)	19 (13, 23)	19 (13, 23)	19 (13, 23)

### 3.2 Immunogenicity evaluation

#### 3.2.1 Immunogenicity set analysis

**Fig. 2:**
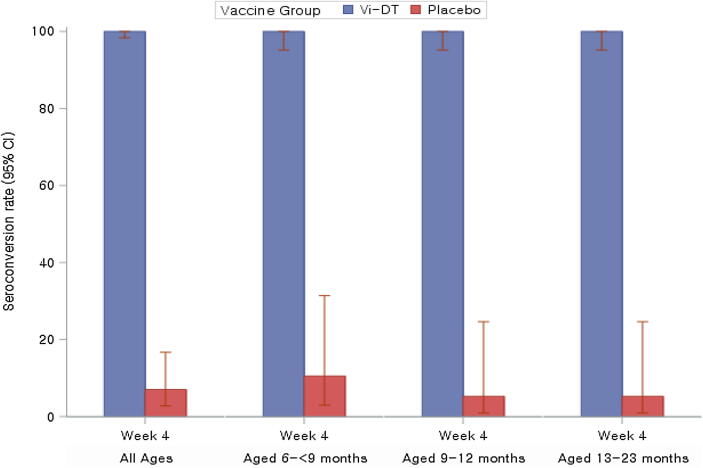
Seroconversion rates as measured by anti-Vi IgG ELISA Response. [Note] Seroconversion rate is proportion of participants who had 4-fold rise in titers compared to baseline (Day 0, Week 0) to post dose. Among all participants the difference in seroconversions post single dose compared to the placebo group was statistically significant (P < 0.0001).

All participants in Vi-DT group showed 100% (95% CI: 98.34, 100.0) seroconversion post single dose vs 7.02% (95% CI: 2.76, 16.70) in the placebo group (P < 0.0001). The seroconversion rate was 100% (95% CI: 95.19, 100.0) in all 3 age strata while rates in placebo recipients were 10.53% (95% CI: 2.94, 31.39), 5.26% (95% CI: 0.94, 24.64), and 5.26% (95% CI: 0.94, 24.64), respectively (Fig. 2, Table 2).

**Table 2:** Seroconversion rates as measured by anti-Vi IgG ELISA.

All Ages:							
Response	Time point		Vi-DT Group		Placebo Group		P-value
**Seroconversion rate**^a^**(95% CI)**	First Dose	Day 0	228	–	57	–	–
		Week 4	228	100.0 (98.34, 100.0)	57	7.02 (2.76, 16.70)	<0.0001

**Age Strata1: 6 to less than 9 m**							
**Response**	**Time point**		**Vi-DT Group**		**Placebo Group**		**P-value**
**Seroconversion rate**^a^**(95% CI)**	First Dose	Day 0	76	–	19	–	–
		Week 4	76	100.0 (95.19, 100.0)	19	10.53 (2.94, 31.39)	–

**Age Strata2: 9–12 m**							
**Response**	**Time point**		**Vi-DT Group**		**Placebo Group**		**P-value**
**Seroconversion rate**^a^**(95% CI)**	First Dose	Day 0	76	–	19	–	–
		Week 4	76	100.0 (95.19, 100.0)	19	5.26 (0.94, 24.64)	–

**Age Strata3: 13–23 m**							
**Response**	**Time point**		**Vi-DT Group**		**Placebo Group**		**P-value**
**Seroconversion rate**^a^**(95% CI)**	First Dose	Day 0	76	–	19	–	–
		Week 4	76	100.0 (95.19, 100.0)	19	5.26 (0.94, 24.64)	–

a Proportion of participants who had 4-fold rise in titers compared to baseline (Day 0, Week 0) to post dose.

**Fig. 3:**
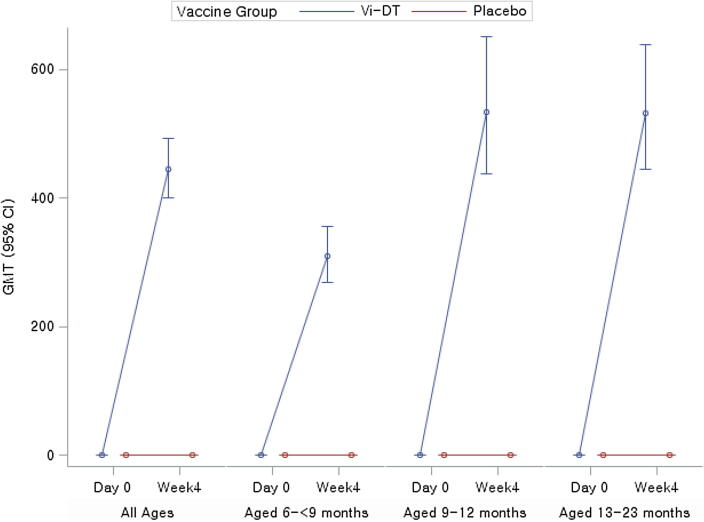
GMT of anti-Vi IgG ELISA response. [Note] Among all participants the difference in GMT comparing Vi-DT to placebo was statistically significant (p < 0.0001).

Four weeks (28 days) post single dose, anti-Vi IgG ELISA GMT were 444.38 IU/ml (95% CI: 400.28, 493.34) and 0.41 IU/ml (95% CI: 0.33, 0.51) in Vi-DT and placebo groups, respectively (P < 0.0001) among all study participants combined. GMT rose from baseline to 28 days post-test dose by 1272.59 fold (95% CI: 1077.26, 1503.32) in Vi-DT recipients while GMT among placebo recipients showed negligible change (0.97 fold, 95% CI: 0.69, 1.35) (p < 0.0001). Looking at age-stratified data, the GMT fold-increase from baseline to 28 days post-test dose was 1006.84 (95% CI: 753.02, 1346.21), 1730.45 (95% CI: 1320.94, 2266.91), and 1182.89 (95% CI: 872.07, 1604.49) in 6 to <9 months, 9 to 12 months, and 13 to 23 months, respectively while the GMT fold-increase in the corresponding placebo groups were 0.74 (95% CI: 0.41, 1.32), 1.10 (95% CI: 0.64, 1.89) and 1.12 (95% CI: 0.61, 2.06), respectively (Fig. 3) (Table 3). Comparing age-stratified groups the differences in GMT fold-increase were statistically significant as two 95% CIs didn’t overlap.

**Table 3:** GMT of anti-Vi IgG ELISA response.

All Ages:							
Response	Time point		Vi-DT Group		Placebo Group		P-value
**GMT**^a^**(95% CI)**	First Dose	Day 0	228	0.35 (0.30, 0.41)	57	0.43 (0.31, 0.59)	0.2792
		Week 4	228	444.38 (400.28, 493.34)	57	0.41 (0.33, 0.51)	<0.0001
**GMFrise**^b^**(95% CI)**	First Dose	Day 0	228	–	57	–	–
		Week 4	228	1272.59 (1077.26, 1503.32)	57	0.97 (0.69, 1.35)	<0.0001

**Age Strata1: 6 to less than 9 m**							
**Response**	**Time point**		**Vi-DT Group**		**Placebo Group**		**P-value**
**GMT**^a^**(95% CI)**	First Dose	Day 0	76	0.31 (0.23, 0.41)	19	0.53 (0.30, 0.93)	–
		Week 4	76	308.80 (268.28, 355.43)	19	0.39 (0.30, 0.51)	**–**
**GMFrise**^b^**(95% CI)**	First Dose	Day 0	76		19	–	–
		Week 4	76	1006.84 (753.02, 1346.21)	19	0.74 (0.41, 1.32)	–

**Age Strata2: 9–12 m**							
**Response**	**Time point**		**Vi-DT Group**		**Placebo Group**		**P-value**
**GMT**^a^**(95% CI)**	First Dose	Day 0	76	0.31 (0.24, 0.40)	19	0.46 (0.28, 0.76)	–
		Week 4	76	533.50 (437.10, 651.16)	19	0.51 (0.34, 0.75)	–
**GMFrise**^b^**(95% CI)**	First Dose	Day 0	76	–	19	–	–
		Week 4	76	1730.45 (1320.94, 2266.91)	19	1.10 (0.64, 1.89)	–

**Age Strata3: 13–23 m**							
**Response**	**Time point**		**Vi-DT Group**		**Placebo Group**		**P-value**
**GMT**^a^**(95% CI)**	First Dose	Day 0	76	0.45 (0.33, 0.62)	19	0.32 (0.17, 0.60)	–
		Week 4	76	532.69 (444.34, 638.60)	19	0.36 (0.25, 0.51)	–
**GMFrise**^b^**(95% CI)**	First Dose	Day 0	76	–	19	–	–
		Week 4	76	1182.89 (872.07, 1604.49)	19	1.12 (0.61, 2.06)	–

a Geometric Mean Titers (unit: IU/ml).

b Geometric Mean Fold rise from baseline (Day 0) to post dose.

### 3.3 Safety evaluation

There was only one immediate reaction among participants. A Vi-DT participant from the 9–12 months of age strata experienced an immediate reaction (erythema/redness and fever) after vaccination; mild in severity and resolved without sequelae.

There was no statistical difference in the proportions of participants who experienced solicited AEs within 7 days between Vi-DT and placebo groups: 25.88% (95% CI: 20.63, 31.93) and 19.30% (95% CI: 11.13, 31.34), respectively and was not statistically significant at 0.05 type 1 error (two sided). Table 4 provides the proportion of participants with solicited AEs by group, by ages combined and individual age strata. Irrespective of group and age strata, pain and tenderness were the most common local solicited AEs; while fever was the most commonly reported systemic AE. Due to the co-administration of MMR, nasopharyngitis was the most commonly reported systemic AE in the 9–12 months age strata and the proportion of participants reported was similar between the Vi-DT (23.68%) and placebo (21.05%) groups. All solicited AEs reported were mild to moderate in severity, except one case of severe fever in a Vi-DT recipient in the 6 to <9 month age stratum that resolved without sequelae.

**Table 4:** Summary of proportion of Solicited AEs.

	Vi-DT Group			Placebo Group			
Within 7 days after first dose	N	Number of participants (%)	95% CI	N	Number of participants (%)	95% CI	P-value
All ages	228	59 (25.88%)	(20.63, 31.93)	57	11 (19.30%)	(11.13, 31.34)	0.295
6 to < 9 month	76	17 (22.37%)	(14.46, 32.93)	19	4 (21.05%)	(8.51, 43.33)	–
9 to 12 months	76	29 (38.16%)	(28.06, 49.40)	19	5 (26.32%)	(11.81 , 48.79)	–
13 to 23 months	76	13 (17.11%)	(10.28, 27.10)	19	2 (10.53%)	(2.94 , 31.39)	–

At 4 weeks, unsolicited AEs were reported by 60.09% (95% CI: 53.61, 66.23) and 66.67% (95% CI: 53.72, 77.51) of participants in the Vi-DT and placebo groups, respectively and was not statistically significant at 0.05 type 1 error (two sided). Table 5 provides a summary of all AEs. All unsolicited AEs reported were of mild to moderate severity, and most of them were assessed unrelated to Vi-DT or placebo administration. During the 28 days post administration, in all age strata, 59 (25.88%) among the Vi-DT and 13 (22.81%) among the placebo recipients had acute respiratory infections. Gastroenteritis was reported by 31 (13.60%) and 16 (28.07%) among the Vi-DT and placebo recipients, respectively. There were no specific safety signals reported from any age strata.

**Table 5:** Summary of AEs.

Within 4 weeks after IP dose (all ages)		Vi-DT Group (N = 228)		Placebo Group (N = 57)	
		Number of AEs	Number of Participants (%)	Number of AEs	Number of Participants (%)
**Solicited AE** (day 0 to day 7)		158	59 (25.88%)	32	11 (19.30%)
Severity:	Mild	131	54 (23.68%)	28	11 (19.30%)
	Moderate	26	19 (8.33%)	3	2 (3.51%)
	Severe	1	1 (0.44%)	1	1 (1.75%)
	Potentially life threatening	0	0 (0.00%)	0	0 (0.00%)
Relatedness:	Definitely Related	17	10 (4.39%)	0	0 (0.00%)
	Probably Related	64	31 (13.60%)	11	5 (8.77%)
	Possibly related	77	29 (12.72%)	21	9 (15.79%)
	Unlikely related	0	0 (0.00%)	0	0 (0.00%)
	Not Related	0	0 (0.00%)	0	0 (0.00%)
**Unsolicited AE**		210	137 (60.09%)	72	38 (66.67%)
Severity:	Mild	201	132 (57.89%)	69	38 (66.67%)
	Moderate	9	8 (3.51%)	3	3 (5.26%)
	Severe	0	0 (0.00%)	0	0 (0.00%)
	Potentially life threatening	0	0 (0.00%)	0	0 (0.00%)
Relatedness:	Definitely Related	0	0 (0.00%)	0	0 (0.00%)
	Probably Related	17	15 (6.58%)	1	1 (1.75%)
	Possibly related	38	36 (15.79%)	12	10 (17.54%)
	Unlikely related	26	23 (10.09%)	10	9 (15.79%)
	Not Related	129	94 (41.23%)	49	31 (54.39%)
**SAE**		1	1 (0.44%)	0	0 (0.00%)

One unrelated SAE with the diagnosis of febrile convulsion secondary to urinary tract infection was reported 5 days after Vi-DT administration in the 6 to <9-month age stratum. It was mild in severity and resolved without sequelae after medical management. No SAEs were reported from the placebo group.

## 4 Discussion

In 2017 the World Health Organization Strategic Advisory Group of Experts (SAGE) on immunization recommended the introduction of TCV for infants and children over 6 months of age as a single dose in typhoid-endemic countries [5]. Vi polysaccharide vaccine cannot be used below 2 years due to lack of T-cell dependent response. For the polysaccharide vaccines, these limitations can be overcome by conjugation of the Vi polysaccharide to a carrier protein. Conjugation of a bacterial polysaccharide to a carrier protein converts the immune response from T-cell independent to T-cell dependent characterized by antibody affinity maturation, IgG subclass switching, and induction of memory [26].

Carrier proteins such as recombinant exoprotein A from *Pseudomonas aeruginosa* (rEPA), tetanus toxoid (TT), diphtheria toxoid (DT) or a non-toxic mutant of diphtheria toxin (CRM 197) have been used successfully for Vi conjugate vaccines [26], [27], [28]. DT is known for its safety profile and is considered as a reliable carrier protein, successfully used for meningococcal conjugate vaccines [29]. Typhoid Vi polysaccharide conjugated to DT as carrier protein was therefore a logical choice for development and Vi-DT had thus been evaluated in phase I clinical trials in the Philippines and Indonesia with potent immune responses in children and adults and no apparent safety concerns [23], [30].

In this Phase II study, Vi-DT showed to be safe, well tolerated, and immunogenic in an important target age group. No subject was withdrawn from the study due to AE. All solicited and unsolicited AEs were mild or moderate in intensity in both the groups and the one SAE was not related to vaccination. Overall, Phase II Vi-DT single dose results confirmed the findings in the Phase I study in the Philippines and in Indonesia, with a safety profile in line with that of other Vi- polysaccharide conjugate vaccines [28], [30], [31], [32], [33], [34].

All subjects in Vi-DT group (100%) seroconverted, with a greater than 4-fold rise in serum anti-Vi IgG 28 days after the initial dose of Vi-DT. Only 7% of placebo recipients seroconverted in the same time period. Similar results were seen within each age stratum. The results from this study are in agreement with other studies of typhoid conjugate vaccines in similar age cohorts.

Vi-TT conjugate vaccine prequalified by WHO was administered as a single dose in infants and toddlers aged 6–23 months and the seroconversion rate (>4-fold rise at day 42 over day 0 baseline) was reported as 98.1% (95% CI: 95.7, 99.2), 97.8% (95% CI: 93.6, 99.5) and 98.2% (95% CI: 94.6, 99.6) in the following age groups respectively, 6–23 months, 6–11 months and 12–23 months [33]. Similar seroconversion rates after single dose Vi-DT were seen: 100% (95% CI: 98.34, 100.0) in all ages in our study. At 42 days post single dose of Vi-TT, anti-Vi IgG GMT fold increases over baseline were 205, 193, and 215 in age strata of 6-23 months, 6–11 months, and 12–23 months, respectively [33] while at day 28 post Vi-DT GMT fold increases of 1272.59, 1006.84, 11730.45, and 1182.89 in age strata 6-23 months, 6–<9 months, 9–12 months and 13–23 months, respectively. Though, immune results from the above mentioned studies can’t be compared & concluded directly because of the different ELISA assay methods used for anti-Vi IgG testing.

These data support the potent immunogenicity and satisfactory safety profile of Vi-DT in 6–23 month-old participants and set the stage for further clinical development of Vi-DT in larger number of participants and broader target populations of 6 months to 45 years of age. The current Phase II study is ongoing at RITM and the final analysis is expected in 2 years.

## Declaration of Competing Interest

The authors declare that they have no known competing financial interests or personal relationships that could have appeared to influence the work reported in this paper.

## References

[1] John J., Van Aart C.J.C., Grassly N.C. (2016). The burden of typhoid and paratyphoid in India: systematic review and meta-analysis. PLoS Negl Trop Dis..

[2] GBD 2017 Typhoid and Paratyphoid Collaborators (2019). The global burden of typhoid and paratyphoid fevers: a systematic analysis for the Global Burden of Disease Study 2017. Lancet Infectious Dis.

[3] Abucejo P.E., Capeding M.R., Lupisan S.P., Arcay J., Sombrero L.T., Ruutu P. (2001 Sep). Blood culture confirmed typhoid fever in a provincial hospital in the Philippines. Southeast Asian J Trop Med Public Health.

[4] GOVPH. Department of Health. 2018 Typhoid Morbidity Week 52. Available at: <https://www.doh.gov.ph/sites/default/files/statistics/2018_TYPHOID_MW52_1.pdf> (Accessed in April 2019).

[5] Background Paper on Typhoid Vaccines for SAGE Meeting (October 2017) <http://www.who.int/immunization/sage/meetings/2017/october/1_Typhoid_SAGE_background_paper_Final_v3B.pdf>. accessed 22nd February 2019.

[6] Crump J.A., Luby S.P., Mintz E.D. (2004). The global burden of typhoid fever. Bull World Health Organ.

[7] Crump J.A., Mintz E.D. (2010). Global trends in typhoid and paratyphoid fever. Clin Infect Dis.

[8] Buttha Z.A., Kliegman R.M., Stanton B.F., Schor N.F., Geme J.W., Berhman R.E. (2011). Salmonella. Nelson Textbook of Pediatrics.

[9] Buckle G.C., Walker C.L., Black R.E. (2012). Typhoid fever and paratyphoid fever: systematic review to estimate global morbidity and mortality for 2010. J Global Health.

[10] Bhutta Z.A., Capeding M.R., Bavdekar A., Marchetti E., Ariff S., Soofi S.B. (2014). Immunogenecity and safety of the Vi-CRM197 conjugate vaccine against typhoid fever in adult, children, and infants in south and southeast Asia: results from two randomised, observer-blind, age de-escalation, phase 2 trials. Lancet Infect Dis.

[11] Owais A., Sultana S., Zaman U., Rizvi A., Zaidi A.K.M. (2010). Incidence of typhoid bacteremia in infants and young children in southern coastal Pakistan. Pediatr Infect Dis J.

[12] Lin F.Y.C., Ho V.A., Khiem H.B., Trach D.D., Bay P.V., Thanh T.C. (2001). The efficacy of Salmonella typhi Vi conjugate vaccine in two-to-five-year-old children.

[13] Ochiai R.L., Acosta C.J., Danovaro-Holiday M.C., Balqing D., Bhattacharya S.K., Agtini M.D. (2008). A study of typhoid fever in five Asian countries: disease burden and implications for controls. Bull World Health Organ.

[14] Gaind R., Paglietti B., Murgia M., Dawar R., Uzzau S., Cappuccinelli P. (2006). Molecular characterization of ciprofloxacin-resistant Salmonella enterica serovar Typhi and Paratyphi A causing enteric fever in India. J Antimicrob Chemother.

[15] Arjyal A., Basnyat B., Nhan H.T., Koirala S., Giri A., Joshi N. (2016). Gatifloxcain versus ceftriaxone for uncomplicated enteric fever in Nepal: an open-label, two-centre, randomised controlled trial. Lancet Infect Dis.

[16] Keddy K.H., Smith A.M., Sooka A., Ismail H., Oliver S. (2010). Fluoroquinolone-resistant typhoid, South Africa. Emerg Infect Dis.

[17] Lunguya O., Lejon V., Phoba M.F., Bertrand S., Vanhoof R., Verhaegen J. (2012). Salmonella Typhi in the Democratic Republic of the Congo: fluoroquinolone decreased susceptibility on the rise. PLoS Negl Trop Dis.

[18] Crump J.A., Karlsson M.S., Gordon M.A., Parry C.M. (2015). Epidemiology, clinical presentation, laboratory diagnosis, antimicrobial resistance and antimicrobial management of invasive Salmonella infections. Clin Microbiol Rev.

[19] Wain J., Hendriksen R.S., Mikoleit M.L., Keddy K.H., Ochiai R.L. (2014). Typhoid fever. Lancet.

[20] Yousafzai MT, Qamar FN, Shakoor S, Saleem K, Kazi M, Garett D, et al. Outbreak investigation of cefriaxone resistant S. Typhi in Hyderabad, Pakistan. 10th International Conference on Typhoid and other Invasive Salmonellosis. April 4–6. Kampala, Uganda, 2017.

[21] Typhoid vaccines: WHO position paper, March 2018 - Recommendations. Vaccine. 2019;37(2):214-216, doi:10.1016/j.vaccine.2018.04.022. 10.1016/j.vaccine.2018.04.022 29661581

[22] Antillón M., Bilcke J., Paltiel A.D., Pitzer V.E. (2017). Cost-effectiveness analysis of typhoid conjugate vaccines in five endemic low- and middle-income settings. Vaccine.

[23] Capeding M.R., Teshome S., Saluja T., Syed K.A., Kim D.R., Park J.Y. (2018). Safety and immunogenicity of a Vi-DT typhoid conjugate vaccine: Phase I trial in Healthy Filipino adults and children. Vaccine.

[24] Sahastrabuddhe Sushant, Saluja Tarun (2019). Overview of the typhoid conjugate vaccine pipeline: current status and future plans. CID.

[25] Rijpkema S., Hockley J., Logan A., Rigsby P., Atkinson E., Jin C. (2018). Establishment of the first International Standard for human anti-typhoid capsular Vi polysaccharide IgG. Biologicals.

[26] Jakobsen H., Jonsdottir I. (2003). Mucosal vaccination against encapsulated respiratory bacteria - New potentials for conjugate vaccines?. Scand J Immunol.

[27] An S.J., Yoon Y.K., Kothari S., Kim D.R., Kim J.A., Kothari N. (2012). Immune suppression induced by Vi capsular polysaccharide is overcome by Vi-DT conjugate vaccine. Vaccine.

[28] van Damme P., Kafeja F., Anemona A., Basile V., Hilbert A.K., de Coster I. (2011). Safety, immunogenicity and dose ranging of a new VI-CRM 197 conjugate vaccine against typhoid fever: Randomized clinical testing in healthy adults. PLoS ONE.

[29] Tontini M., Berti F., Romano M.R., Proietti D., Zambonelli C., Bottomley M.J. (2013). Comparison of CRM197, diphtheria toxoid and tetanus toxoid as protein carriers for meningococcal glycoconjugate vaccines. Vaccine.

[30] Medise B.E., Soedjatmiko S., Rengganis I., Gunardi H., Sekartini R., Koesno S. (2019). Six-month follow up of a randomized clinical trial phase I study in Indonesian adults and children: safety and immunogenicity of Salmonella typhi polysaccharide-diphtheria toxoid (Vi-DT) conjugate vaccine. PLoS ONE.

[31] Mitra M., Shah N., Ghosh A., Chatterjee S., Kaur I., Bhattacharya N. (2016). Efficacy and safety of vi-tetanus toxoid conjugated typhoid vaccine (PedaTyphTM) in Indian children: School based cluster randomized study. Hum Vaccines Immunother.

[32] Canh D.G., Lin F.Y., Thiem V.D., Trach D.D., Trong N.D., Mao N.D. (2004). Effect of dosage on immunogenicity of a Vi conjugate vaccine injected twice into 2- to 5-year-old Vietnamese children. Infect Immun.

[33] Mohan V.K., Varanasi V., Singh A., Pasetti M.F., Levine M.M., Venkatesan R. (2015). Safety and Immunogenicity of a Vi Polysaccharide-Tetanus Toxoid Conjugate Vaccine (Typbar-TCV) in Healthy Infants, Children, and Adults in Typhoid Endemic Areas: A Multicenter, 2-Cohort, Open-Label, Double-Blind, Randomized Controlled Phase 3 Study. Clin Infect Dis.

[34] Chinnasami B., Mangayarkarasi V., Prema A., Sadasivam K., Davis M.J. (2013). Safety and Immunogenicity of Salmonella Typhi Vi conjugate vaccine (Peda TyphTM) in children upto five years. Int J Sci Res Publ.

